# Technologies for Beneficial Microorganisms Inocula Used as Biofertilizers

**DOI:** 10.1100/2012/491206

**Published:** 2012-04-01

**Authors:** E. Malusá, L. Sas-Paszt, J. Ciesielska

**Affiliations:** ^1^Research Institute of Horticulture, 96-100 Skierniewice, Poland; ^2^CRA, Centre for Plant-Soil Systems, Turin, Italy

## Abstract

The increasing need for environmentaly friendly agricultural practices is driving the use of fertilizers based on beneficial microorganisms. The latter belong to a wide array of genera, classes, and phyla, ranging from bacteria to yeasts and fungi, which can support plant nutrition with different mechanisms. Moreover, studies on the interactions between plant, soil, and the different microorganisms are shedding light on their interrelationships thus providing new possible ways to exploit them for agricultural purposes. However, even though the inoculation of plants with these microorganisms is a well-known practice, the formulation of inocula with a reliable and consistent effect under field conditions is still a bottleneck for their wider use. The choice of the technology for inocula production and of the carrier for the formulation is key to their successful application. This paper focuses on how inoculation issues can be approached to improve the performance of beneficial microorganisms used as a tool for enhancing plant growth and yield.

## 1. Introduction

Environmental issues such as freshwater pollution, energy saving, and soil erosion are forcing the farmers to introduce methods of cultivation that have a lower impact on the environment. The application of environmentaly friendly practices is promoted by voluntary certification schemes (e.g., GlobalGAP or organic farming schemes) as well as by legally binding regulations (e.g., the EU Directive 2009/128 aiming at the implementation of sustainable pest management practices). In this context, the reduced use of chemical fertilizers with increased application of organic fertilizers is considered a compulsory route to alleviate the pressure on the environment derived from agricultural practices.

Several organic fertilizers have been introduced in the recent years, which are also acting as natural stimulators of plant growth and development [1, 2]. A specific group of this kind of fertilizers includes products based on plant growth-promoting microorganisms (PGPM). Three major groups of microorganisms are considered beneficial to plant nutrition: arbuscular mycorrhizal fungi (AMF) [3], plant growth-promoting rhizobacteria (PGPR) [4], and nitrogen-fixing rhizobia, which are usually not regarded as PGPR [5]. Microbial inoculants based on these microorganisms can be divided into different categories depending on their use, even though exact definition of these categories is still unclear. Nevertheless, the category of biofertilizer most commonly refers to products containing soil microorganisms increasing the availability and uptake of mineral nutrients for plants (like rhizobia and mycorrhizal fungi). According to the definition proposed by Vessey [6], biofertilizers are substances which contain living microorganisms which, when applied to seed, plant surfaces, or soil, colonize the rhizosphere or the interior of the plant, and promote growth by increasing the supply or availability of primary nutrients to the host plant. Another category of PGPM-containing products is that of phytostimulators which are generally containing auxin-producing bacteria, inducing root elongation [7].

The interest in the application of these products is rising due to the enhancement in nutrient uptake efficiency [8, 9] and society demands for more green technologies in production [10], increasing costs of agrochemicals. Furthermore, biofertilizers and phytostimulators possess secondary beneficial effects that would increase their usefulness as bioinoculants. Indeed microorganisms such as *Rhizobium* and *Glomus* spp. have been shown to also play a role in reducing plant diseases [11].

The practice of inoculating plants with PGPM can be traced back to the early 20th century, when a product containing *Rhizobium *sp. was patented (Nobbe and Hiltner 1896, cited in [12]). Mycorrhizal fungi, even though utilized as biofertilizers since few decades, were reported to promote plant growth through P uptake since the late 1950s [13]. Since then, research efforts in these fields have steadily increased, resulting, in recent years, in the selection of numerous strains showing several beneficial features [4, 6, 13–16].

The policies supporting sustainable agricultural production and extensive research that has improved the effectiveness and consistency of microbial inocula have resulted in the registration of several strains for both biocontrol [17] and biofertilization [4], with mycorrhizal and PGPR preparations being marketed in several countries. Yet, a wider use of microbial inoculants, especially those acting as phytostimulators and biofertilizers, has been frequently hampered due to the variability and inconsistency of results between laboratory, greenhouse, and field studies. The reason for these discrepancies lies in the incomplete understanding of the complex relationships established between the components of the system: the plant, the microorganisms, and the environmental conditions, particularly that of soil [18]. In addition, the lack of correct formulations and the expensive and time-consuming procedures of registration are also among the factors holding back the use of PGPM on a wider scale [19].

The present paper is focusing at different issues related to formulation of inoculants to improve the performance of PGPM use in agriculture, particularly for the purpose of plant nutrition.

## 2. Inoculation Technology

PGPM inoculants can be defined as formulations containing one or more beneficial microorganism strains (or species) prepared with an easy-to-use and economical carrier material. The development of techniques for the production of large quantities of pure inocula, with high infectivity potential, is the main issue to be tackled in order to allow a wide use of biofertilizers. The key aspects in PGPM inoculation technology are the use of a proper formulation of inocula preparations, the selection of an adequate carrier, and the design of correct delivery methods.

### 2.1. Inocula Formulation

The production of selected bacteria and yeasts in pure cultures is a quite common practice making use of fermenters. Therefore, once the particular strain/s for the inoculum have been selected, an industrial standardized process of production can be defined [20]. However, in case of biofertilizers, unlike that of biopesticides, the cost of production is an important constraint considering that the price of the fertilizer shall not exceed that of conventional ones to assure a market sustainability. Hence, several cheap organic matrixes (e.g., whey, water sludges, composts, etc.) have been tested as growth media for PGPM [21, 22]. Another approach to reduce the production costs is by using agroindustrial residues enriched with rock phosphate. During composting or fermentation, free or immobilized microorganisms that produce organic acids are added to the matrix, improving the solubilization of phosphate, which make it more available to plants [23].

Recently, the use of biofilms has also been proposed as possible means to produce effective plant inocula [24]. A biofilm consists of microbial cells embedded into a self-produced polymeric matrix (known as an extracellular polymeric substance—EPS) and adherent to an inert or living surface, which provides structure and protection to the microbial community. Three major types of biofilms can occur in the soil: bacterial (including *Actinomycetes*), fungal, and fungal-bacterial biofilms. Both bacterial and fungal biofilms are formed on abiotic surfaces, while fungi act as the biotic surface in formation of fungal-bacterial biofilms [24]. The majority of plant-associated bacteria found on roots and in soil are forming biofilms [25]. Therefore, using PGPM strains that are forming biofilms could be a strategy to ease the formulation and production of inocula. Furthermore, biofilm-based inocula could also facilitate the production of biofertilizers considering the biofilm as a carrier (see below).

While ectomycorrhizal fungi can be produced under fermentation conditions, the production of AMF inocula poses several difficulties due to the need of a plant host for the multiplication of the mycorrhizal fungi. The first attempts in AMF inocula production used pot cultures with soil mixtures, or other technologies (such as aeroponics) [13, 26]. However, the development of monoxenic cultures in the late 1980s [27, 28] has allowed the production of AMF under strictly controlled conditions. A method utilizing split-plate cultures and Ri T-DNA transformed roots of carrots [29] was developed to produce spores. Even though the method allows a higher efficiency with production on average of 15.000 spores per Petri dish 4-5 months after beginning the production cycle, it has been used mainly for physiological and laboratory studies. The improvement of this method proposed by Douds [30] requires replacing the media in the distal compartment every 2 months and concomitantly replenishing the carbon source in the proximal compartment with glucose. The results are a production of about 65.000 spores in 7 months. Yet, such methods are mainly used for the production of batches of spores for trials or for maintenance of genebanks since the annual cost for producing one spore was estimated to be up to 30–50 USD, depending on the method utilized [31]. Recently, a large-scale *in vitro* production of mycorrhizal fungi, feasible for implementation on a commercial scale, has been proposed [32]. It emphasizes the selection of appropriate Ri T-DNA transformed host roots for different AMF species, the choice and maintenance of the growth medium, and the application of quality assurance procedures.

However, commercial inoculants containing AMF species are still produced mainly by growing host plants in controlled conditions, with the inclusion in the inoculant of different fungal structures (spores, mycelium hyphae) and containing mycorrhizal roots residues from the plants used as the propagating material (i.e., sorghum, maize, onion, or *Plantago lanceolata*). This could be considered a classical method where substrates of sand/soil and/or other materials (e.g., zeolite, perlite) are used to mass-produce AM fungal inoculum in pots, bags, or beds, for large-scale applications. Critical aspects of this production method are [33]

- the use of known AMF species [34],
- the choice of the host species with a short life cycle, adequate development of the root system, a good colonization level by a large range of AM fungi, and tolerance to relatively low levels of phosphorus,
- the manipulation of mineral nutrients level in soil,
- the appropriate combination of AMF species and host plant.

With this technique, it is possible to reach inoculum densities of 80–100 thousand propagules per litre [35]. This implies the need of diluting the inoculum with a carrier for the preparation of a commercial product (see below).

Considering that microbial associations between bacteria and mycorrhizal fungi have been observed to occur naturally in the soil promoting mycorrhizal symbiosis [36, 37], enhanced formulations could include two or more species of different PGPM. Microbial consortia can stimulate plant growth through a range of mechanisms that improve nutrient acquisition and inhibit fungal plant pathogens [18, 38]. The different mechanisms proposed to explain such growth stimulation relate to the increased rate of nutrients cycling due to enhanced soil microbial content and microorganisms biodiversity found in soil where mycorrhizal plants are grown [37, 39].

Simultaneous inoculation with different PGPR and/or AMF often resulted in increased growth and yield, compared to single inoculation through improved nutrient uptake [40, 41]. Indeed, the interactions between bacteria and AM fungi have beneficial functions related to nutrient uptake, particularly when PGPR [42–44] and N_2_-fixing bacteria [45, 46] are involved.

Inoculation of maize and ryegrass with *A. brasilense* and AMF resulted in N and P contents comparable to plants grown with fertilizer [8]. Coinoculation with different AMF species is generally more likely to be effective due to the general not specificity of AMF fungi colonization of specific plant species/cultivars [47, 48]. Synergistic interaction between AM fungi and several PGPR, including *Azospirillum*, *Azotobacter*, *Bacillus*, and *Pseudomonas* species, has also been reported as beneficial for plant growth (see also the review by Barea et al. [49]). Increased root colonization by AMF was observed when mycorrhizal fungi were coinoculated with PGPR [50, 51]. Four times higher nodule number was observed when plants were inoculated with a mixture containing *Glomus deserticola *and *Rhizobium trifoli*, in comparison to single *R. trifoli*, inoculation, and enhanced mycorrhization and nodulation was obtained with coencapsulated *R. trifoli *and *Yarrowia lipolytica *[52]. Inoculation with nodule-inducing rhizobia and AM fungi resulted in increasing both P and N uptake efficiency [53]. Mycorrhizal and nodule symbioses often act synergistically on infection rate, mineral nutrition, and plant growth [54]. Coinoculation resulted in enhanced uptake of mineral nutrients and increased growth [55, 56] also when PGPM were applied as commercial biofertilizers containing consortia of different microorganisms [9, 57–60].

All these examples are pointing to the usefulness and higher efficacy of biofertilizers composed by more species having different mechanisms of growth promotion. The availability of several strains of PGPR [15] and AMF [33] tested in different crops species and under different field conditions should allow the definition of consortia suitable for commercial uses.

### 2.2. Carriers

The carrier is the major portion (by volume or weight) of the inoculant that helps to deliver a suitable amount of PGPM in good physiological condition [61]. The materials constituting the carrier can be of various origins: organic, inorganic, or synthesized from specific molecules. Availability and cost are the main factors affecting the choice of a carrier.

The carrier should be designed to provide a suitable microenvironment for the PGPM and should assure a sufficient shelf life of the product (at least 2-3 months for commercial purposes, possibly at room temperature). The formulation should allow an easy dispersion or dissolution in the volume of soil near the root system. A good carrier should therefore posses as much as the following properties: good moisture absorption capacity, easy to process and free of lump-forming materials, near-sterile or easy to sterilize by autoclaving or by other methods (e.g., gamma-irradiation), low cost and availability in adequate amounts, and good pH buffering capacity [62]. For carriers that shall be used for seed coating, a good adhesion to seeds is also important [63]. Other characteristics that are affecting the carrier appropriateness are a standardized composition ensuring chemical and physical stability, suitability for as many PGPM species and strains as possible, the possibility of mixing with other compounds (i.e., nutrients or adjuvants), and being composed of biodegradable and nonpolluting compounds [61]. In case the inoculant is used as seed coating, the carrier shall assure the survival of the PGPM on the seed since normally seeds are not immediately sown after seed coating [64]. Survival of the PGPM is important both during the storage period of the bioproduct and after being introduced into the soil [65]. The latter is fundamental for defining the application technology and dosing the product: the inoculant has to compete with native soil microorganisms for the nutrients and habitable niches, and has to survive against grazing protozoa [66, 67]. Carrier materials that make available nutrients and/or habitable micropore to the PGPM, particularly in case of bacteria, would then be more suitable.

The kind of carrier utilized defines the physical form of the biofertilizer. Dry inoculants can be produced using different kinds of soil materials (peat, coal, clays, inorganic soil), organic materials (composts, soybean meal, wheat bran, sawdust, etc.), or inert materials (e.g., vermiculite, perlite, kaolin, bentonite, silicates) [61]. Liquid inoculants can be based on broth cultures, mineral or organic oils, or on oil-in-water suspensions.

In the case of solid carriers, powder, granules, or beads are the most typical forms utilized. Standard sizes of the powder material may vary from 75 *μ*m to 0.25 mm [61]. The size of granules and beads ranges from 100–200 *μ*m to 3-4 mm in diameter [12]. Powder-type inoculants can be used to coat seeds or suspended in a liquid to form a slurry that is directly applied to the furrow or, alternatively, the seeds/plants are dipped in it just prior to sowing/planting [68].

Bacteria can also be stored by lyophilization, which allows achieving high survival rates [69], without any carrier. However, during the process a cryoprotectant must be added, which is essential for protecting the bacterial cell membrane and cytoplasm against dehydration. Mannitol is a good protectant, but recently microcrystalline cellulose has also proved useful due to its slower degradation kinetics in soil and the high stability of the inoculum at room temperature for a long period [70]. Lyophilized microbial cultures can be incorporated into a solid carrier or utilized directly.

The addition in the formulation of carbon sources (e.g., skimmed milk) [71] or stimulatory compounds (e.g., substances present in soil organic matter) that could increase the efficiency of inoculation is also another issue that could be considered while designing a formulation. *G. intraradices* hyphal growth and root inoculation were increased by specific organic matter components (humic acids or fractions fraction enriched in structures chemically related to 3,4,5-trihydroxybenzoic acid or syringic acid) [72, 73]. Treating seeds with a formulation of *B. subtilis* AF 1 in peat supplemented with chitin or chitin-containing materials showed better control of different soil-borne pathogens and enhanced plant growth than the bacteria culture alone [74].

#### 2.2.1. Natural Carriers

Peat has been commonly used as a carrier for PGPR, particularly for rhizobia inoculants, due to its wide availability and a long history of field trials [75]. When added to peat, PGPR maintain metabolic activity and in some cases can continue to multiply during the storage period, thus increasing their population size, but this can vary with different stains [75]. However, a major drawback of peat is the variability in its quality and composition (acidity), due to its origin from different production sites, which can affect PGPM viability [76]; furthermore, peat holds a large load of microorganisms, which can reduce the shelf life of the inoculant [77]. Similar disadvantages can be listed for carriers made of plant waste materials, which are generally not used for commercial preparations. On the other hand, composts could also be considered as possible carriers, especially when the process of their production involves the use of specific selected strains. For example, adding N-fixing and P-solubilizing bacteria to a vermicompost increased the amount of N and phosphorus availability in the final product [23, 78]. However, composted organic materials are not always useful as AMF carriers. Cellulose-rich amendments could reduce the mycorrhization rate in the case of not fully composted materials [79, 80] even though cellulose can increase the asymbiotic hyphal growth of AMF [81]. Sawdust was shown to be useful as a carrier for production of inocula containing different strains of bacteria [82].

The increasing availability of sludge wastewater has led to consider also this material as a growth medium and carrier for PGPM inoculants. A sludge with heavy metals content below the legal limits was safely used in production of bacterial inoculants [22]. Sludge-based carrier maintained desired rhizobia populations (10^7^-10^8^ cells g^−1^), with pH around neutral and an acceptable water holding capacity, after 130 days of storage at 25°C [83].

Coal, clays, and inorganic soils (i.e., lapillus, volcanic pumice or diatomite earths) are available in different regions and can be used as carriers [75]. Their microbial load depends on the site of production (about 10^2^-10^3^ CFU g^−1^), but it is generally lower than in organic carriers. Vermiculite, perlite, and bentonite are also available in different countries, but their use is generally limited due to the difficulty in creating a formulation [84]. Indeed, the effect of these carriers on bacteria viability and mobility is dependent on the pH, ion strength, and the electrolyte in solution [85]. Expanded clay has been tested as a carrier for AMF [86], and mycorrhized roots mixed with soil are also the simplest AMF inocula. Among other inorganic compounds, glass beads have also been proposed for AMF inocula [87]. A mixture of organic and inorganic materials have been proved successful in increasing activity and shelf life of Burkholderia sp [88].

All of the above mentioned carriers rely on the absorption of the microorganisms by the substance/matrix of the carrier. This method of inclusion has some drawbacks, particularly in relation to the survival of the microorganisms and their protection during transport, storage, and handling. Nevertheless, some processes with different carriers using such approach have been patented:

- the Belgian patent no. 521.850 for use of diatomaceous earth and colloidal silica for Rhizobium,
- the British patent no. 1.777.077 for the use of bentonite for Rhizobium,
- French Patent no. 1.180.000 using a must juice, to which substances with an adsorbing action are added, such as cellulose, bone meal, kaolin, or silica gel, in the manufacture of preparations rich in bacteria of the Azotobacter group,
- United States Patent no. 4956295 for the stabilization of dried bacteria extended in particulate carriers, where dried viable bacteria are mixed in a particulate carrier composed primarily of an inorganic salt of low moisture absorbing capacity together with a minor proportion of a silica gel absorbent. The inorganic salts may be sodium or calcium carbonates, bicarbonates, sulfates, or phosphates.

#### 2.2.2. Polymer-Based Carriers

The increased interest in the application of bacterial preparations as plant protection products has promoted studies aiming at improving their stability and increasing their shelf life. However, the same approach could be successfully applied to products containing PGPRs and AMF. Among the new materials utilized as carriers for PGPM, organic polymers have been evaluated. These are compounds (e.g., polysaccharides) that in the presence of ions or by changing chemical conditions (e.g., a change in pH of the medium) form cross-links that create a complex structure. The polymers encapsulate, or “immobilize”, the microorganisms in the matrix and release them gradually through a degradation process. Polymer formulations offer a long shelf life even at ambient temperature since they provide protection against environmental stresses and a consistent batch quality due to standardized production. Nevertheless, storage at cool temperature (4°C) allows to maintain a longer viability of encapsulated cells [12]. These inoculants can be added or mixed with nutrients to improve the survival of the bacteria upon inoculation.

Alginate, a natural polymer of D-mannuronic acid and L-glucuronic acid, is the most commonly used substance for microbial cell encapsulation. It is derived mainly from brown macroalgae such as *Macrocystis pyrifera* (kelp), but recently also another macroalga (*Sargassum sinicola*) has been shown to produce alginate of similar physical characteristics [89]. The reaction between alginate and a multivalent cation (e.g., Ca2^+^) forms a gel consisting of a dense three-dimensional lattice with a typical pore-size range of 0.005 to 0.2 mm in diameter [90]; when the alginate solution is dropped into the cation solution beads are formed. Alginate beads generally have a diameter of 2-3 mm, but microbeads with a size of 50 to 200 *μ*m that can entrap up to 10^8^ to 10^9^ CFU g^−1^ have also been proposed [91].

Inclusion of bacteria in alginate beads has been utilized for different species, either spore forming and non sporulating [12]. Different AMF structures have also been entrapped into alginate matrixes [92, 93] or in beads formed with different polymers [94]. Spores of mycorrhizal fungi were entrapped in alginate film formed in a PVC-coated fibreglass screen [95], and roots of leek seedlings inoculated with this alginate film containing *G. mosseae* spores were heavily colonized after few weeks of growth in greenhouse conditions. Similar results were obtained with spores obtained from monoxenic cultures embedded into beads [96]. Inclusion of filamentous fungi such as Aspergillus [97] and Actinomycetes has been also proved possible (Malusá, Trzczyński and Taddei, unpublished observations).

Alginate beads can maintain a sufficient amount of live cells to assure inoculation up to several months [65, 98]. However, improving the viability of inocula is still an issue. To tackle it, several approaches have been tested. Adding nutrients (e.g., skimmed milk) to the inoculum [70] or freeze-drying gel beads in presence of glycerol [99] resulted in a prolongation of beads shelf life. Intraradical structures of* G. intraradices* embedded in alginate beads were still infective after up to 62 months after storage in plastic vials at 4°C [100]. However, it shall be considered that freeze-drying of alginate beads can result in some collapse of the matrix [101]. Therefore, the addition of fillers (material added to the moulding mixture to reduce cost and/or improve mechanical properties) should be considered when planning this technological process. Adding chitin to the beads [102] helped preserve their porous cellular structure resulting in significantly higher porosity values when compared to starch filled beads [103] and resulted in higher bacterial efficacy when evaluating their effect on plants. Addition of 0.5% kaolin to freeze-dried alginate-glycerol beads significantly increased bacterial survival also under UV light radiation [104].

Reducing the cost of the production process and enhancing the physical characteristics of the beads were also obtained by encapsulation and air-drying of bacteria into a mixture made of alginate (3%), standard starch (44.6%), and modified starch (2.4%) [105]. This process allowed to obtain beads that after drying have a water content of 7%, size of 4 mm, and a mechanical resistance of about 105 Newton (features similar to that of grain seeds). Storage at room temperature or at 4°C did not affect the viability of the encapsulated bacteria, which were able to survive up to six months maintaining a final population size of about 10^8^ CFU g^−1^ (corresponding to about 10^5^ CFU bead^−1^) [106]. However, with this composition, some problems can arise when standardizing and automating the beads formation due to the viscosity of the mixture and the need of a continuous agitation of the stock medium (Malusá and Wawrzyńczak, unpublished observations). Recently, a process using starch industry wastewater as a carbon source for the production of *Sinorhizobium meliloti* with simultaneous formulation using alginate and soy oil as emulsifier has been proposed, showing a cell viability of more than 10^9^ CFU mL^−1^ after 9 weeks of storage [106]. Addition of synthetic zeolite to the alginate mixture did not improve the survival of the embedded microbial cells, nor the physical structure of the beads [65].

Other polymers have been tested with AMF. Carragenan was used to encapsulate AMF structures while hydroxyethylcellulose was used as a gel carrier [107]. Two patents have also been registered:

- French Patent application no. 77.10254 (corresponding to U.S. Patent no. 4.155.737) which makes use of a polymer gel based on polyacrylamide gel or a silica gel for different microorganisms,
- the US patent 5021350 on the process for inclusion of mycorrhizae and actinorhizae in a polymer gel matrix based on at least one polymer from the polysaccharide group, with at least partial cross-linking of the polymer.

#### 2.2.3. Promising New Technologies

Water-in-oil emulsions appear to be a good, yet underutilized, method for storing and delivering microorganisms through liquid formulations [108]. The oil traps the water around the organism and, therefore, slows down water evaporation once applied. This is particularly beneficial for organisms that are sensitive to desiccation or in case of the use for horticultural crops where irrigation systems are in place. Water-in-oil emulsions allow the addition of substances to the oil and/or aqueous phases which could improve both cell viability and release kinetics. However, cell sedimentation during storage is a major issue to be considered. Studies are carried out aiming at solving this problem with the help of nanomaterials. Thickening the oil phase using hydrophobic silica nanoparticles significantly reduced cell sedimentation and improved cell viability during storage [109].

Recently, a new process based on the application of supercritical fluid properties has been tested to encapsulate virus formulations [110] and could also be applied to prepare bacterial inocula. The process, named PGSS (Particles from Gas Saturated Solutions), is carried out at low temperatures and uses carbon dioxide as a supercritical fluid. Therefore, there should be no negative effects on the microorganisms' viability, and the cost of production would be relatively cheap. The final product of the process is almost spherical particles that form a free-flowing powder which can be suspended in water [111]. The possibilities of the PGSS process have already successfully been demonstrated for several solids and liquids [111, 112].

Another interesting new technology is proposing the exploitation of the natural production of bacterial biofilms as a possible carrier, and not only for the production of the inoculum, of defined bacterial or fungi-bacteria consortia. Biofilm production is already obtained for different industrial applications (e.g., wastewater treatment, production of chemical compounds) [113]. Two types of biofilms are employed in that case: biofilms growing onto inert supports (charcoal, resin, concrete, clay brick, sand particles) and biofilms that are formed as a result of aggregate formation. In the first case, biofilms grow all around the particles, and the size of the biofilm particles grows with time usually to several mm in diameter. Biofilm formed by aggregation is called granular biofilm; granule formation may take from several weeks to several months [113].

There are four stages to the development of a mature biofilm: initial attachment, irreversible attachment by the production of EPS, early development, and maturation of biofilm architecture [114]. Particularly critical is the production of EPS, which serves to bind the cell to the surface and to protect it from the surrounding environment. EPS can be composed of polysaccharides, proteins, nucleic acids, or phospholipids. A common EPS produced by bacterial cells in biofilms is the exopolysaccharide alginate [115].

The speed of biofilms formation and maturation is affected by surface, cellular, and environmental factors. Rough surfaces, porous, and less hydrophobic materials tend to enhance biofilm formation [116]. Biofilms tend to form more readily in the presence of optimum nutrients availability, particularly of phosphorous which increase the adhesion ability of cells [117]. High temperature increases the rate of cell growth, EPS production, and surface adhesion, all of which enhance biofilm formation [118]. Biofilm reactors can be assembled in a number of configurations including batch, continuous stirred tank, packed bed, trickling bed, fluidized bed, airlift reactors, upflow anaerobic sludge blanket, and expanded bed reactors [113].

Beneficial biofilms developed in *in vitro* cultures containing both fungal and bacterial strains were used as biofertilizers for nonlegume species with good efficacy results [24]. Application of a biofilmed inoculant containing a fungal-rhizobia consortium significantly increased N_2_ fixation in soybean compared to a traditional rhizobium inoculant [119]. Wheat seedlings inoculated with biofilm-producing bacteria exhibited an increased yield in moderate saline soils [120]. Biofilms seem also to help the microorganisms to survive after inoculation even under stress conditions: this is a key aspect for the effectiveness of PGPM inoculation under agricultural conditions. Inocula made with biofilms were shown to allow their rhizobia survive at high salinity (400 mM NaCl) by 105-fold compared to rhizobial monocultures [24]. Interestingly, beneficial endophytes were observed to produce higher acidity and plant growth-promoting hormones than their mono- or mixed cultures with no biofilm formation [121].

Technologies used for the production of living hybrids materials could be a new frontier in the development of carriers for PGPMs. Silica has appeared as a promising host for microorganisms encapsulation: immobilization pathways are based on immobilization of population bacteria dispersed into a silica gel. Bacteria can be either entrapped into alginate microbeads coated with silica membranes or into macrocavities created inside the silica matrix. Such material improves the mechanical properties of the alginate bead, reduces cell leakage, and enhance cell viability [122].

The application of bionanotechnologies could also provide new avenues for the development of carrier-based microbial inocula [123, 124]. Nanotechnology employs nanoparticles which are made of inorganic or organic materials, that are defined by having one or more dimensions in the order of 100 nm or less [125]. The integration of whole cells with nanostructures leads to hybrid systems that have numerous applications in many fields including agriculture [126]. Indeed, even though nanoscale constructs are smaller than cells, macroscopic filters, made of radially aligned carbon nanotube walls, able to absorb *Escherichia coli*, were fabricated [127]. The same technology could therefore be applied to collect bacterial cells from fermentation processes and deliver them to the plant. The physical stability and the high surface area of nanotubes, together with the ease and cost-effective fabrication of nanotube membranes may thus expand their use in the production of biofertilizer.

The use of nanoformulations may enhance the stability of biofertilizers and biostimulators with respect to dessication, heat, and UV inactivation. The addition of hydrophobic silica nanoparticles of 7–14 nm to the water-in-oil emulsion formulation of the biopesticide fungus *Lagenidium giganteum* reduced the desiccation of the mycelium. The physical features of the formulation were improved and the microorganism was still effective after 12 weeks of storage at room temperature [109].

## 3. Application Methods

A limited array of methods exists for the delivery of PGPM to crops in the field. Farmers are not keen on purchasing specialized equipment to be used for microbial-based products. Therefore, formulated inocula should be readily applied using standard farming machinery and straightforward methods. Inoculation can be done through application to the plant material or to the soil. The latter method can be more convenient for the farmer because of less time required, but generally a higher amount of inoculant is then needed. Soil inoculation can be done either with solid or liquid formulations. Normally, the inert material is mixed with the inoculum in the factory, but it could be mixed by the farmer prior to application, especially when liquid formulations are used. The use of fertilizers which were produced mixing organic matrixes and insoluble phosphates with addition of selected P-solubilizing microorganisms can also be considered a method to apply PGPM to crops: it increases the availability of nutrients (particularly of P) to plants and eventually affects the tolerance of the plant to soil pathogens [128].

Application methods depend on the kind of crop concerned: annual crops can be inoculated by broadcasting the inoculum over the soil surface, alone or together with seeds, or by in-furrow application, seed dressing, or coating; tree crops can be initially inoculated by root dipping or seedling inoculation [64]. Application to already established orchards or plantations can present some technical problems, when inocula have to be distributed to the soil [129]. The need to deliver the PGPM as close as possible to the root system can be fulfilled by liquid formulations applied through a fertigation system. Trials using alginate beads and polyurethane foam as carriers to deliver PGPM into the water solution are showing the feasibility of this approach (Malusá, Trzcinski and Treder, unpublished observations); however, there is the need to either soak the foam for some time into the water tank or dissolve the beads using a citrate solution. Alternatively, powder materials can be buried near the roots using a harrow-like device associated with a distributor. Subsequent irrigation would increase the transfer of the inocula toward the roots and may enhance the efficacy of the inoculation, creating better moisture conditions which favor the movement of the bacteria in soil [130].

Microbial populations in the soil could dilute or counteract the effect of introduced PGPM. In the case of PGPR, the recovery of the inoculated strains in the soil or on root rhizosphere was limited to 30–40 days after inoculation [66]. Therefore, repeated applications (3-4) during the growing season, with an interval of 2–4 weeks, increase the effectiveness of PGPM applications.

## 4. Conclusions

A better understanding of the different conditions and features of the interrelationships in the soil-plant-microorganism system is needed to improve the efficacy of PGPM inocula applications in the field. Indeed, particularly for bacteria, many factors are involved in determining their rhizocompetence. Together with the genotype and physiological state of the inoculated strain, the size and composition of the populations sustained by the rhizosphere is determined by several environmental factors: soil pH, mineral nutrients, and water content; species, genotype, and physiological state of the plant; the presence of other microbial species [131].

Several isolates have been obtained in the last decades showing plant growth enhancement or biocontrol properties. Yet the knowledge of PGPM behaviour at the root level and their function in the field environment is still limited [36, 37].

Considering the beneficial effects of PGPR and AMF, studies using inoculant mixtures are opening a new approach to the subject. These studies would facilitate the designing of large consortia of inocula that bring about a synergistic promotion of plant growth or have multitasking features [132]. However, such mixtures are more technically demanding, since they increase the difficulties in designing a proper inoculant that would fit the different strains and kinds of microorganisms. The designing of biofilmed-based carriers [24] or of encapsulation techniques which allow the production of macrocapsules consisting of a core and an envelope could facilitate the development of biofertilizers formed of microbial consortia.

Most inoculants selected to date have been designed for annual crops (mainly legumes, cereals, and some vegetables). However, there is an increasing demand from other agricultural sectors such as fruit and vegetable production, and particularly from organic farming and integrated production systems, where synthetic inputs are not allowed or their use is limited by legal restrictions. Soilless and protected crops can also be an interesting market for commercial inoculants, where the predictability of PGPM applications should be higher than in open fields due to the use of inert substrates and controlled growth conditions. The selection of specific strains for all these crops can further expand the market for inocula and support the shift in agriculture toward more sustainable production systems. However, also the development of technologies aiming at an efficient delivery of the inocula, probably by modification of sprayers and sprinklers normally used for plant protection or irrigation, could further increase the use and enhance the reliability of PGPM applications under agricultural conditions.

The future challenges in selecting PGPM are related to the attempts at alleviating abiotic stress conditions in crops (i.e., drought, salinity, inorganic, and organic pollutants) and improving food quality. Improvements in the production process for consortia of microbial inocula, the development of new carriers based on nanoparticles, optimization of application devices and of the time of application for polyannual crops, are all issues requiring further research to widen the implementation and efficient use of PGPM in agriculture. Such need is also prompted by the policy decisions supporting sustainable practices, as well as by the reassessment of the safety of plant protection agents, currently underway both in EU and USA, which can further foster the market potential for PGPM.
